# Study characteristics and methods in a national nursing and allied health research programme: A descriptive study

**DOI:** 10.1111/inr.70007

**Published:** 2025-02-22

**Authors:** Jules Barbier, Laurent Poiroux, Julien Bouix‐Picasso, Sonia Guillouet, Valérie Berger, Anne‐Sylvie Ramelet, Judith Leblanc

**Affiliations:** ^1^ Hôpital Ambroise Paré Hôpitaux de Paris (AP‐HP) Boulogne‐Billancourt France; ^2^ Centre Hospitalier Universitaire d'Angers Faculté de santé de l'Université d'Angers Angers France; ^3^ Laboratoire Éducations et Promotion de la Santé Université Sorbonne Paris Nord Villetaneuse France; ^4^ Faculté des sciences infirmières Université de Montréal Montréal Quebec Canada; ^5^ Académie de santé des armées École du Val‐de‐Grâce Service de santé des armées Paris France; ^6^ CHU de Caen Normandie Pôle de Formation et de Recherche en Santé de l'Université de Caen Anticipe U1086 Caen France; ^7^ Centre Hospitalier Universitaire de Bordeaux CeDS UR 7440 Université de Bordeaux Talence France; ^8^ Faculté de biologie et médecine Suisse Institute of Higher Education and Research in Healthcare, Faculty of biology and medicine Institut universitaire de formation et de recherche en soins Université de Lausanne Lausanne University Lausanne Switzerland; ^9^ Centre Hospitalier Universitaire Vaudois Suisse Lausanne University Hospital Lausanne Switzerland; ^10^ Sorbonne Université INSERM Institut Pierre Louis d’Epidémiologie et de Santé Publique, Assistance Publique ‐ Hôpitaux de Paris (AP‐HP), Hôpital St Antoine, Clinical Research Platform Paris‐East Paris France

**Keywords:** mixed methods, nursing, policy; nursing policy, research; evidence‐based practice, research; health services research, research; qualitative methods, research; quantitative methods, research; research dissemination, research; research evaluation, research; research methods

## Abstract

**Aim:**

To describe the characteristics, methods and publications of studies funded by a French research programme for nursing and allied health professions.

**Background:**

In some countries, the emerging field of nursing and allied health research is supported by funding programmes that are poorly documented.

**Design:**

Observational descriptive study.

**Methods:**

Data from funded studies in 2012–2020 were identified in publications, registries or an institutional repository by two authors in April–May 2022. Methods (quantitative, qualitative, mixed‐method), study designs using the mixed‐methods appraisal tool and thematic categories were extracted.

**Results:**

In total, 212 studies were funded. The yearly number of studies has risen from 19 in 2012 to 33 in 2020. Eight nursing and allied health professions were represented among grant recipients, including 42% (89/212) of nurses. Half of the studies with identified methods were multicentric (91/185, 16% in 2012 and 71% in 2018). The median funding per study has risen from €63,471 in 2012 to €251,665 in 2020. The predominant methods were quantitative and interventional (144/185, 78%), with randomised controlled trials being the most frequent study design (125/144, 87%). Among the 38 quantitative observational studies, 14 (37%) were diagnostic studies. Three qualitative or mixed‐methods studies were reported. The thematic categories included rehabilitation, nursing care, complementary medicine, patient education and metrology. Of the 50 completed studies, 18 publications were identified.

**Conclusions:**

The number of studies, multicentric studies and funding appeared to increase over the nine‐year study period. The predominance of high‐level evidence studies is encouraging, although a limited number of studies have been published.

**Implications for nursing and health policy:**

This study highlights the gradual involvement in a nursing and allied health research programme and the support needed to disseminate research.

## INTRODUCTION

To efficiently meet complex healthcare needs, nursing and allied health professionals are ethically required to use up‐to‐date evidence‐based knowledge (International Council of Nurses, 2021; World Physiotherapy, 2022). High‐quality and rigorous research is the most effective way to generate relevant knowledge to inform practice.

Research funding is necessary to conduct studies that meet international standards for good clinical and research practice. However, obtaining funding is difficult for many researchers, and this is particularly a concern for nurses and allied health professionals, who must compete with more established professions (Kim et al., 2022). There is a lack of specific funding agencies for these professions, and when funding allocated to research is available, the amount is often insufficient. A study suggested that in the United States, the National Institute of Health budget would need to increase fivefold to adequately fund nursing research (Kiely & Wysocki, 2020).

In Europe, the European Union (EU) is investing in research, innovation and implementation to address current healthcare challenges through two major programmes: EU4Health and Horizon Europe. These programmes aim to foster European healthcare researchers’ collaboration and support ambitious initiatives to improve the health of European populations (European Commission, 2021). Nevertheless, evaluations of the number of successful grant applications by nurses and allied health professionals are not available, either at the European or national level (Kim et al., 2022).

In France, a national research grant programme, ‘Programme Hospitalier de Recherche Infirmière et Paramédicale (PHRIP)’, was created in 2009 for nurses and was extended to allied health professions the following year (French Ministry of Health, 2009). Its objective is to support clinical research aimed at guiding nursing and allied health practice. The programme, further referred to as the French research programme for nursing and allied health professions, is the first and unique national funding programme of clinical research for these professions. It enables these professionals to be grant recipients and conduct studies from conception to publication. Being a nurse or an allied health professional according to French regulation was a condition for eligibility, except from 2013 to 2015, when the eligibility criteria were extended to nursing and allied health practice in a broader sense. During this period, any healthcare professional could apply.

## BACKGROUND

In many countries, governments have recognised the need to develop research capacity for nurses and allied health professionals. In most Western countries, health research is funded via national interdisciplinary structures that focus on health problems (e.g., the Canadian Institutes of Health Research or the National Institute for Health and Care Research in the United Kingdom) or types of funding, such as hospital or university funding (Eckert et al., 2023). In North America, the first National Centre for Nursing Research was created in 1985 (known today as the National Institute of Nursing Research).

To meet international quality standards, research programmes have developed evaluation criteria. The National Institutes of Health and many other funding agencies evaluate the methodological approach, investigator profile, significance, innovation and environment. These criteria are useful indicators for monitoring projects, particularly in the context of an emerging field that is nursing and allied health research, as in France. However, the impact of research programmes for nurses and allied health professionals on patients and families and, more broadly, on healthcare services, policy, and society is poorly documented (Hilder et al., 2020; Hulcombe et al., 2014; Kim et al., 2022). Above all, it is very complex to identify precisely the amounts of funding obtained by research nurses and allied health professionals. This may be explained by the fact that a large amount of funding is interdisciplinary, by a lack of interest in organising the collection of profession‐specific data, and by a lack of more general hindsight on the allocation of funding. In the absence of such data, it is not possible to assess the impact of the research funds obtained. The overall number of publications in nursing, a common indicator of the success of research programmes, seems to have increased worldwide (Zhu et al., 2021). However, there are considerable differences in funding access and research activity between countries (Wang et al., 2022).

Overall, the number and the nature of the research grants obtained by nurses and allied health professionals are poorly evaluated (Kim et al., 2022), and, when evaluated, the level of evidence of the studies is limited. An analysis of publications in the top 10 nursing journals between 2000 and 2006 showed that approximately half of the funded studies were observational and descriptive in nature (Mantzoukas, 2009). Nursing studies published between 1985 and 2010 were mostly observational (Yarcheski et al., 2012). Similarly, among 1600 articles in physiotherapy journals, a few were interventional studies, and 13% of all studies were randomised controlled trials (Paci et al., 2009). More recently, Richards et al. reported low proportions of high‐quality experimental studies in nursing, which limits the production of high‐level evidence to inform clinical practice (Richards et al., 2018). Given this context, describing the nursing and allied health research that is funded and evaluating the methods and study designs selected is crucial. These evaluations serve to describe and analyse both methodological strengths and limitations of funded projects. The overarching goal is to promote rigorous research whose results can directly be implemented into patient care practices with measurable healthcare outcomes.

In France, a descriptive analysis of the number of funded studies conducted five years after the research programme for nursing and allied health professions was launched highlighted the need for greater methodological support for researchers but did not evaluate the research methods and study designs used (Stuwe et al., 2015). No further analyses of the grant programme have been performed since.

### Aim of study

The study aimed to describe the characteristics, research methods and publications of the studies funded by the French research programme for nursing and allied health professions.

## METHODS

### Research design and eligibility criteria

This retrospective descriptive observational study included all studies selected by the French research programme for nursing and allied health professions from 2012 to 2020. The Ministry of Health provides public detailed data on funded studies only since 2012. The end of the selection criteria in 2020 allowed for a sufficient timeframe for data collection in 2022. No exclusion criteria were applied.

### Outcomes

The main outcomes included the overall proportion of methods, classified as quantitative, qualitative, or mixed‐method, and the study designs of the funded studies. Data were extracted from a combination of publications in peer‐reviewed journals, study declarations in international or French clinical trial registries, or study titles reported by the funder in an institutional repository.

The methods and study designs were defined using a tool selected prior to the start of data collection, the mixed‐methods appraisal tool (MMAT) (Hong et al., 2018). The MMAT facilitates the evaluation process by providing, in a single tool, methodological quality criteria for quantitative, qualitative and mixed‐methods studies.

Other outcomes were (1) the study characteristics, including the proportions of professions represented among grant recipients, their position as principal investigator or scientific leader, their gender and academic background, the proportion of French regions represented, and the median funding allocated overall and per year; (2) the yearly proportions of methods and the associated study designs; (3) the thematic categories; and (4) the description of the published studies, including the methodological quality using the MMAT, the author rank of the grant recipient and the journal characteristics.

### Data collection

The study title, acronym, grant recipient, allocated funding, institution managing the funding and study number assigned were extracted from a public institutional repository of the French research programme for nursing and allied health professions, available on the Ministry of Health website (https://sante.gouv.fr/systeme‐de‐sante/innovation‐et‐recherche/).

Then, the method, the type of study design and, where applicable, the type of randomisation, blinding and number of participating sites were extracted from the sources in the following order of priority: (1) publications in electronic databases, including the MEDLINE, Web of Science and Cochrane databases; (2) clinical trial registers; or (3) study titles reported by the grant programme. The order was determined based on the availability of data, their accuracy and their completeness.

In order to define the profile of the grant recipient and to describe the geographical distribution of the funded studies, the profession and gender of the grant recipient, his or her academic training following initial healthcare training, and the French region of the institution managing the funding were collected using data reported in the registries, the National Directory of Health Professionals or a standard search engine.

The profession has been classified using a list of 13 nursing and allied health professions covered by French regulations, as follows: nurses, physiotherapists, podiatrists, occupational therapists, psychometricians, speech and language pathologists, orthoptists, radiologic technologists, medical technologists, hearing aid practitioners, opticians, prosthetists and dietitians. Other professional categories could be selected, such as physicians, psychologists and others.

### Data extraction from the clinical trial registries

All studies were searched in the ClinicalTrials.gov register using the programme's public data. Data were retrieved from the following sections of the register*: study design, arms/groups/cohort, study description and principal investigator*.

In the absence of data on the number of centres involved in the above sections, data from the *Locations* section were used.

For randomised controlled trials, randomisation was considered individual in the absence of specific information.

Studies without an up‐to‐date project status in the register were considered completed once their results were published.

If necessary, the search was pursued in the CENTRAL register, the EU Clinical Trials Register, the International Clinical Trials Registry Platform and in French trial registries from ‘Assistance Publique—Hôpitaux de Paris (AP‐HP)’ and ‘Institut National du Cancer’.

### Data extraction from the publications

For studies listed in ClinicalTrials.gov, publications were searched using referencing in ClinicalTrials.gov and in the MEDLINE, Web of Science and Cochrane databases using the name of the grant recipient or the grant programme (PHRIP).

The journals’ impact factors and publication domains were extracted using Clarivate^TM^ 2020. The domain with the highest ranking was selected.

The data extraction was conducted by a single author (JB) when the type of study was clearly stated in the study title or in the other sources of data. Studies with unclear information were reviewed by a second independent expert (JL, LP, JBP, or SG), who also used MMAT criteria. Any disagreements were resolved by consensus.

The data extraction was performed in April and May 2022 using a predefined list of items validated by the author group (Supporting Information Table S1) and integrated into an Excel file. Studies with missing data were not excluded. Missing data were reported.

### Data analysis

The analysis used descriptive statistics. Categorical variables were expressed as numbers and proportions, and continuous variables were expressed as numbers or medians and the first quartile and third quartile, further referred to as interquartile ranges (IQRs). Analyses were performed using R studio, v4.1.2 software (R Core Team, 2022).

The type of study design and the methodological quality of publications were defined using the MMAT. This tool was developed with international experts to enable the critical evaluation of the quality of articles and to distinguish methods. In this study, four potential study designs were added a priori: a single‐case experimental design, diagnostic study, trial within cohort and randomised controlled trial based on care data.

### Thematic categories

From the study titles, the population, the intervention, the comparator, the outcome measures (PICO), and the clinical specialty were identified when possible. Using the method of content analysis by Mayring, deductive and inductive content analyses were used to create categories (Mayring, 2000). Deductive analyses were guided by the standard PICO. Titles were analysed and coded in an Excel file by two independent authors (ASR and VB), who performed formative reliability checks throughout the process. The frequencies of the results were tabulated as a final step.

### Ethical considerations

The Direction of Clinical Research and Innovation of the AP‐HP, the institution of the first and last authors, consulted on 4 January 2022, declared the study in conformity with the European General Data Protection Regulation without the need to request additional authorisation to conduct the study and present its results.

## RESULTS

### Characteristics of the studies

A total of 212 studies were funded by the French research programme for nursing and allied health professions from 2012 to 2020, with a median per year of 22 studies (IQR: 19–28). Other than a decrease in 2017, the number of studies per year has risen from 19 in 2012 to 33 in 2020 (Supporting Information Figure S1).

Grants were awarded to professionals from 8 of the 13 nurses and allied health professions covered in France (8/13, 62%) as well as to physicians or psychologists (Table 1). The professionals most frequently represented were nurses (89/212, 42%) and physiotherapists (44/212, 21%).

**TABLE 1:** Main characteristics of the studies.

	*N* (%)
Profession of the grant recipient	*N* = 212
Nurse^a^	89 (42.0%)
Physiotherapist^a^	44 (20.8%)
Speech and language pathologist^a^	13 (6.1%)
Dietitian^a^	11 (5.2%)
Occupational therapist^a^	9 (4.2%)
Radiologic technologist^a^	8 (3.8%)
Physician	6 (2.8%)
Orthoptist^a^	3 (1.4%)
Psychomotrician^a^	1 (0.5%)
Psychologist	1 (0.5%)
Not available	27 (12.7%)
Gender of the grant recipient	*N* = 212
Female	148 (69.8%)
Not available	1 (1.1%)
Region	N = 212
Ile‐de‐France	52 (24.5%)
Auvergne‐Rhône‐Alpes	34 (16.0%)
Occitanie	31 (14.6%)
Nouvelle‐Aquitaine	19 (9.0%)
Bretagne	18 (8.5%)
Pays de la Loire	17 (8.0%)
Provence‐Alpes‐Côte d'Azur	10 (4.7%)
Grand Est	9 (4.2%)
Normandie	9 (4.2%)
Centre‐Val de Loire	7 (3.3%)
Hauts‐de‐France	4 (1.9%)
Bourgogne‐Franche‐Comté	2 (0.9%)
Status	*N* = 212
Ongoing	83 (39.2%)
Completed	50 (23.6%)
Suspended or stopped	8 (3.8%)
Not available	71 (33.5%)

^a^ These professions are classified among the 13 nursing and allied health professions under French regulations.

Overall, 70% (148/212) of grant recipients were women, including 73% (65/89) of women among nurse grant recipients and 50% (22/44) of women among physiotherapist grant recipients.

One‐third of the grant recipients declared additional academic training, including 30 doctorates (30/212, 14%).

In the registers, the grant recipient was the coordinating investigator or scientific director in 65% of the cases (73/113 studies with available data) (Supporting Information Table S2). In the other studies, when the names specified were not those of the grant recipient, two‐thirds of the coordinating investigators were physicians (27/40, 68%).

The studies came from 12 of France's 18 regions, particularly the Paris metropolitan area (52/212, 25%), the Auvergne–Rhône–Alpes region (34/212, 16%) and the Occitanie region (31/212, 15%).

The total allocated funding for the grant programme was €39,434,131. The funding allocated was €1,260,496 in 2012 and €8,516,729 in 2020 (median per year: 3,721,021; IQR: 2,849,797–5,635,853). The median funding per study has risen from €63,471 in 2012 to €251,665 in 2020 (Supporting Information Figure S2).

Overall, 50 studies had a completed status (50/212, 24%), 83 were ongoing (39%), and 8 were declared suspended or stopped (4%) (Table 1).

### Methods and study designs

The research method was extracted for 185 of the 212 studies (87%). For the remaining 27 studies, no data on methods were found in the registries or in the study titles, and no publication was identified. A total of 41 studies (41/185, 22%) were evaluated by two independent reviewers because of study design ambiguities (30/41, 73%), incomplete forms (6/41, 15%) or complex designs (5/41, 12%).

A quantitative interventional method was used in 78% of the studies (144/185), and a quantitative observational method was used in 21% (38/185) (Table 2). A qualitative method was used for two other studies, and a mixed‐method method was used for the remaining study.

**TABLE 2:** Study methods.

	*N* (%)
Methods^a^	*N* = 185
Quantitative interventional	144 (77.8%)
Quantitative observational	38 (20.5%)
Qualitative	2 (1.1%)
Mixed	1 (0.5%)
Interventional study designs	*N* = 144
Randomised controlled trial (including randomised cross‐over study)	125 (86.8%)
Diagnostic study	5 (3.5%)
Non‐randomised trial	5 (3.5%)
Before‐and‐after study	4 (2.8%)
Time series	3 (2.1%)
Single‐case experimental design	2 (1.4%)
Observational study designs	*N* = 38
Diagnostic study	14 (36.8%)
Cohort study	9 (23.7%)
Incidence study	6 (15.8%)
Case series	4 (10.5%)
Cross‐sectional study	2 (5.3%)
Survey	2 (5.3%)
Ecological study	1 (2.6%)
Randomisation	*N* = 125
Individual	112 (89.6%)
Cluster stepped‐wedge	7 (5.8%)
Cluster parallel	4 (3.3%)
Cluster cross‐over	2 (1.6%)
Centres	*N* = 185
Multicentric^b^	91 (49.2%)
Not available^c^	18 (9.7%)

^a^ Methods and study designs were defined according to the MMAT (Hong et al., 2018).

^b^ Number of multicentric studies per year: 3/19, 15.8% (2012), 6/20, 30.0% (2013), 11/28, 39.3% (2014), 11/22, 50.0% (2015), 12/18, 66.7% (2016), 9/14, 64.3% (2017), 17/24, 70.8% (2018), 13/34, 38.2% (2019), 9/33, 27.3% (2020). The number of multicentric studies per year among studies reported in registries was 3/15, 20.0% (2012), 6/18, 33.3% (2013), 11/26, 42.3% (2014), 11/22, 50.0% (2015), 11/14, 78.6% (2016), 9/13, 69.2% (2017), 16/22, 72.7% (2018), 11/28, 39.3% (2019), 5/5, 100.0% (2020).

^c^ Data were missing in 2 studies in 2012, 1 in 2013, 1 in 2015, 1 in 2016, 2 in 2017, 5 in 2019 and 6 in 2020.

Among the studies with a quantitative interventional method, six distinct study designs were identified. Of these studies, 87% (125/144) were randomised controlled trials. The type of randomisation was mainly individual (112/125, 90%). Other studies used cluster randomisation (i.e., cluster, cluster stepped‐wedge or cluster cross‐over).

Among the studies that adopted a quantitative observational method, seven distinct study designs were identified and approximately one‐third were diagnostic studies (14/38, 37%).

The study designs used in the qualitative studies were grounded theory and qualitative description. In the mixed‐method study, the design was convergent.

Overall, randomised controlled trials were the most frequent study design (125/185, 68%).

The studies were all prospective or cross‐sectional; no retrospective studies were found. A total of 49% of the studies were multicentric (91/185), representing 16% (3/19) of the studies in 2012 and 71% (17/24) in 2018.

**FIGURE 1:**
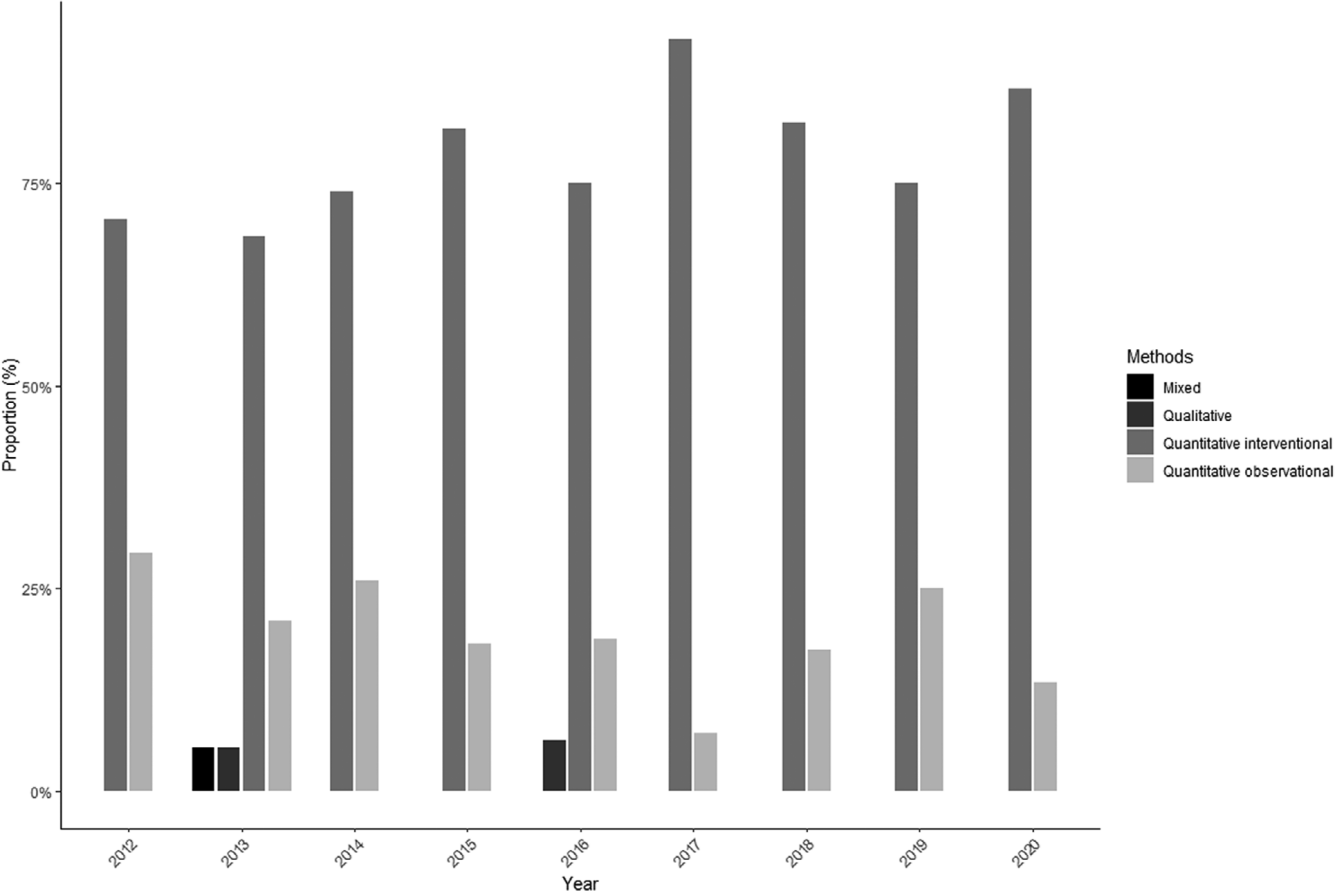
Methods. The methods were defined according to the MMAT (Hong et al., 2018).

Each year, the quantitative interventional method accounted for more than two‐thirds of the studies, ranging from a minimum of 68% in 2013 to a maximum of 93% in 2017 (Figure 1).

Among studies with a quantitative interventional method, randomised controlled trials constituted the majority each year, representing at least 67% of interventional study designs in 2012 and up to 100% in 2020 (Supporting Information Figure S3).

Among studies with a quantitative observational method, no specific study design was found each year (Supporting Information Figure S4).

The year 2019 showed particular designs, with five parallel cluster randomised trials out of a total of 13 cluster trials for the entire study period (38%) as well as two ‘single‐case experimental designs’.

### Thematic categories

Of the 212 studies, we were able to identify five categories from 197 studies (93%). The most frequent category was related to rehabilitation (73/197, 37%), such as respiratory and motor physiotherapy, speech therapy, occupational therapy and psychomotor therapy. The second most frequent category was nursing care (29/197, 15%), which included perioperative support, therapeutic alliance, family caregivers, professional roles and care modalities. The third involved complementary medicine (25/197, 13%), which included hypnosis, musculoskeletal manipulations, sophrology and nonmedicinal therapies. The fourth category was patient education (19/197, 10%). The fifth category was metrology (17/197, 9%), including the development and validation of assessment tools. Finally, topics were related to anaesthesia and critical care procedures (6), training (4), mental health (4), digital tools (3), nutrition (3), prevention (3), organisation of health care (2), sexuality (1), bariatric surgery (1), breastfeeding (1), child development (1), wound care (1), sleep (1), endovascular treatment (1), neurological assessment (1) and ethics (1).

### Publications

In total, 24 studies were published, including 6 research protocols and 18 articles that presented findings (24/212, 11%) (Supporting Information Table S2). The 18 publications represented 36% of the studies with completed status (18/50) and had obtained funding from 2012 to 2016.

Of the 18 studies published, 14 could be assessed for methodological quality. Three other studies were diagnostic studies that could not be evaluated using the MMAT, and one could not be obtained in its full version. The evaluated studies adopted qualitative (*n* = 1), quantitative interventional (*n* = 9) and quantitative observational methods (*n* = 4).

The qualitative studies met all the quality criteria. Among the nine randomised controlled trials, six studies did not meet the criterion of blinding of the evaluation. Of the four observational studies, only one study did not meet one criterion due to a lack of description of the risks of bias.

In 16 of the 18 publications of findings, the grant recipient was an author of the article and was the first author in 10 articles (10/18, 56%). The median impact factor of the journals was 5.8 (IQR: 2.7–7.3), which included high‐impact journals such as the *International Journal of Nursing Studies, the Journal of the American Medical Association* and *the European Respiratory Journal*. The most common area of publication was nursing (7/18, 39%), followed by various clinical specialties.

## DISCUSSION

National research funding programmes on care practices for nursing and allied health professionals are rare and poorly documented (Kim et al., 2022). In a French research programme for these professions, the number of funded studies and multicentric studies appeared to increase over a nine‐year period, as did the funding per study. Eight nursing and allied health professions were represented, with most studies coordinated by nurses. Quantitative interventional methods, particularly randomised controlled trials, were most frequently used. Thematic categories included rehabilitation, nursing care, complementary medicine, patient education and metrology. To date, more than one‐third of the completed studies have been published.

### Progressive involvement

In the French research programme involving 13 nursing and allied health professions, professionals from eight professions participated (Supporting Information Figure S5), with further room for improvement with the involvement of the remaining five allied health professions. The increasing trend in the number of funded studies, their size and the annual funding allocated over the study period highlight the commitment to this programme. Total funding was approximately €40 million between 2012 and 2020 (i.e., 1.8% of the research funds allocated by the French Ministry of Health in 2012 and 6.8% in 2020). Funding for other French calls for projects existing since 1992 and mainly dedicated to physicians (called ‘PHRC’, ‘PHRC‐I’ or ‘PHRC‐K’) over the period 2012–2020 was €906 million, i.e., 23 times greater. Funding for nursing and allied health research increased 7‐fold, while the increase of other funding was minimal (1.7) over the same period. To address the funding disparity related to historical precedents and policy decisions, it is essential for nurses and allied health professionals to proactively pursue grant opportunities, thereby increasing their research capabilities and future representation in funded projects.

### Methods

In the French programme for nursing and allied health professions, grant recipients mainly proposed quantitative interventional studies. Nurses and allied health professionals largely adopted the randomised controlled trial design, which is classically acknowledged as having the highest level of evidence among interventional quantitative approaches. A large proportion of grant recipients came from hospitals where medical research is well established and where randomised controlled designs are widely used. In the present study, the cluster randomisation method, although a minority, gained importance with an annual presence since 2016, in line with the trend observed in clinical research (Arnup et al., 2016).

The gradual increase in the number of multicentric studies funded by the French programme encourages greater generalisability, applicability and dissemination of the findings. Similar to the field of clinical research, which has increased substantially in recent decades, over 50% growth in research output and publications in the nursing field was observed between 2000 and 2019, particularly in English‐speaking Western countries (Yanbing et al., 2021). Our findings underscore the interest in methodologically sound research to guide clinical practice and are encouraging in the international context where high‐quality studies are performed. However, as noted in a recent review, there are still few studies of this type (Eckert et al., 2023).

In French‐funded studies, the reporting of methods according to international standards was heterogeneous. For some studies, no data on study design were found in the registries or a second review was necessary due to lack of clarity.

While the studies were mainly based on well‐known experimental designs, some lesser‐known designs were found, such as the ‘single‐case experimental design’, which is emergent. The large proportion of diagnostic studies representing more than a third of observational designs indicates that professionals are keen to develop new measurement instruments.

Qualitative and mixed‐method approaches were poorly represented in the French research programme, despite advantages such as a complete view of the phenomenon studied and the diversity of data sources (Larue et al., 2009). This can be explained by numerous factors, including a lack of familiarity with these approaches by the grant recipients and methodologists, who may be more influenced by medical research worldviews, or by the members of the jury and experts who assess the grant applications. These results may be related to a lower methodological quality of the studies, which could lead to a low selection rate, and may underline the influence of scientific currents after positivism in health disciplines. Irvine et al. also reported little high‐quality mixed‐method research in the nursing literature (Irvine et al., 2020). More generally, the diversity of methods and study designs should be encouraged, providing that adequate research infrastructure and expertise are given.

### Place of nurses and allied health professionals

In this study, the grant recipient names could not be found in one‐third of the trial register declarations. In two‐thirds of these cases, the investigating coordinator was a physician. These results may suggest reduced participation, whether desired or not, of nurses and allied health professionals after obtaining funding.

Grant recipients may also have been physicians. From 2013 to 2015, the French research programme has evolved, no longer targeting professionals but their practices. The coordination of studies by physicians under this programme could be due to the perceptions by physicians that the programme could fund their own research, or that allied health professionals were not experienced enough to lead the study. Another hypothesis could be linked to French regulations, which stipulate that studies involving a certain level of risk must be conducted exclusively by a physician. The risk is that the research programme will no longer meet the needs of nursing and allied healthcare. We, therefore, urge that the eligibility criteria continue to empower the healthcare professions targeted by the grant programme.

Barriers to research by these professionals are well known in France or worldwide and include clinical workload pressures that result in limited research time as well as a lack of sustained training and guidance (Decullier et al., 2021; Harris et al., 2020).

In terms of gender equity, the number of females among nursing grant recipients appeared to be lower than in the French nursing workforce (73% of nurse grant recipients were women vs. 87% in the French nursing workforce), in accordance with the literature (Feral‐Pierssens et al., 2021). The distribution seemed similar among the physiotherapist workforce and grant recipients from this profession (51% vs. 50%, respectively) (DREES, 2022).

### Involvement at the publication stage

The dissemination of results was considered limited, with 11% of funded studies resulting in publications, 8% of funded studies resulting in the publication of the main findings, and 36% of completed studies published.

In an older French research programme intended primarily for physicians, 30% of funded studies resulted in publications, 17% resulted in the publication of the main findings, and 67% of completed studies were published (French Ministry of Health, 2024, unpublished). These figures are not far removed from those observed in the programme for nursing and allied health research.

It was not possible to assess the reasons why completed studies had not yet been published. For recent studies, manuscripts may be in the process of being written or evaluated by a journal. The limited dissemination of results may be linked to the lesser experience of the professionals involved, as this was often their first study coordination. Possible publication and publication delay biases for studies that do not confirm their main hypothesis may also be mentioned. Other explanations may relate to the position of nurses and allied health professionals in the hospital hierarchy and health research (Eckert et al., 2023; Thompson & Clark, 2012; Yanbing et al., 2021; Zhu et al., 2021).

Language can be a barrier, as most scientific journals in nursing and allied healthcare are in English (Raffing et al., 2021). This is an issue because many funding agencies require open access to the results of funded studies for maximum impact. Developing a funding instrument to support open‐access dissemination of the results could be helpful.

In the present study, grant recipients contributed, as first or last authors, to half of the publications presenting results. Their essential participation at this stage should be encouraged. The journals selected were mainly in the nursing field, which made it possible to target the readership. The publications evaluated had few methodological limitations, including non‐compliance with the blinding criterion which is common in nursing and allied health research (Eckert et al., 2023).

Recent developments in France, with the creation of university commissions in 2019 and academic positions in nursing and rehabilitation sciences, will undoubtedly provide leverage for the dissemination of results and foster greater legitimacy for promoting large‐scale research. In this context, the existence of dedicated research funds contributed to the construction of disciplines.

### Limitations

First, results depended in part on the data provided by project leaders and methodological contributors when the study was entered in the trial registries. The data may have been missing or unclear at the registration stage and could not be completed due to the limited number of publications. However, the expert review minimised bias in interpretation. Second, all registry declarations and publications may not have been identified. Third, the years 2009–2011 of the grant could not be used because they were not sufficiently detailed in the public data. Additional work on the subsequent research trajectories of the grant recipients, the characteristics of studies that are submitted but not accepted, and the application of findings in clinical practice and policy, would also be highly instructive. For this study with a French national dimension, we used the French definition of allied health professions established by national regulations. This may limit the generalizability of our results in an international context. Finally, the thematic analysis was carried out using a quantitative approach only, which probably does not sufficiently describe the topics explored and their diversity.

### Implications for nursing and health policy

While national programmes promoting nursing and allied health research are essential, they are still rare and their evaluation, particularly regarding study methods, remains underdeveloped.

The use of high‐level of evidence study designs must be continued to guide clinical practice, as is encouraged internationally. However, this should not impede other study designs to respond to relevant questions and address the existing research‐practice gap.

System‐level drivers, such as funding initiatives, are essential contributors to the promotion of research. These drivers need to be evaluated and strengthened to encourage the participation of nurses and allied health professionals in research efforts.

Providing evidence that these investments are beneficial to health and well‐being may encourage governments and grant‐awarding bodies to increase funding for nursing and allied health research. As pointed out by Kim et al., the appointment of nurses and allied health professionals to committees that define national research funding policies and priorities, and to funding award panels, seems essential in this respect (Kim et al., 2022).

Research funding and support for training will, together, increase the number of research projects, as well as the knowledge and skills of nursing and allied health researchers to compete successfully for major grants. Ultimately, this will broaden the scope and importance of their research and facilitate the dissemination of the usefulness of their work. Finally, actively supporting the dissemination of the findings through publications appears crucial for promoting the application of research into practice.

## CONCLUSIONS

The description of the studies selected by a national research programme for nursing and allied health professions highlights progressive involvement in research over a nine‐year period and the predominance of high‐level evidence studies, mostly randomised controlled trials. Some of the funded studies have been published. These findings offer perspectives for the promotion of research within the fields of nursing and allied health care.

## AUTHOR CONTRIBUTIONS

*Study design*: Laurent Poiroux, Julien Bouix‐Picasso, Sonia Guillouet, Valérie Berger and Judith Leblanc. *Data collection*: Jules Barbier and Judith Leblanc. *Data analysis*: Jules Barbier, Laurent Poiroux, Julien Bouix‐Picasso, Sonia Guillouet, Valérie Berger, Anne‐Sylvie Ramelet and Judith Leblanc. *Study supervision*: Judith Leblanc. *Manuscript writing*: Jules Barbier, Laurent Poiroux, Julien Bouix‐Picasso, Sonia Guillouet, Valérie Berger, Anne‐Sylvie Ramelet and Judith Leblanc. *Critical revisions for important intellectual content*: Jules Barbier, Laurent Poiroux, Julien Bouix‐Picasso, Sonia Guillouet, Valérie Berger, Anne‐Sylvie Ramelet and Judith Leblanc.

## CONFLICT OF INTEREST STATEMENT

The authors declare that they have no known competing financial interests or personal relationships that could have appeared to influence the work reported in this paper.

## FUNDING INFORMATION

No external funding.

## Supporting information



Supporting Information
