# Supplementary material for: Study characteristics and methods in a national nursing and allied health research programme: A descriptive study
Source: Int Nurs Rev. 2025 Feb 22;72(1):e70007. doi: 10.1111/inr.70007 (PMC11845934; doi:10.1111/inr.70007)
Supplement: Supplementary file 1 — Supporting Information [file INR-72-0-s001.docx]

**Study characteristics and methods in a national nursing and allied health research program: a descriptive study**

**eSupplement**

[eFig. 1. Number of studies in the French research programme for nursing and allied health professions 2](#_Toc171689630)

[eFig. 2. Allocated fundings per study 3](#_Toc171689631)

[eFig. 3. Interventional study designs 4](#_Toc171689632)

[eFig. 4. Observational study designs 5](#_Toc171689633)

[eFig. 5. Proportion of grant recipients among the mean workforce identified over the period in the nursing or allied health professions 6](#_Toc171689634)

[eTable 1. Predefined list of items for data extraction and response modalities 8](#_Toc171689635)

[eTable2. Additional characteristics of the studies 9](#_Toc171689636)

# eFig. 1. Number of studies in the French research programme for nursing and allied health professions


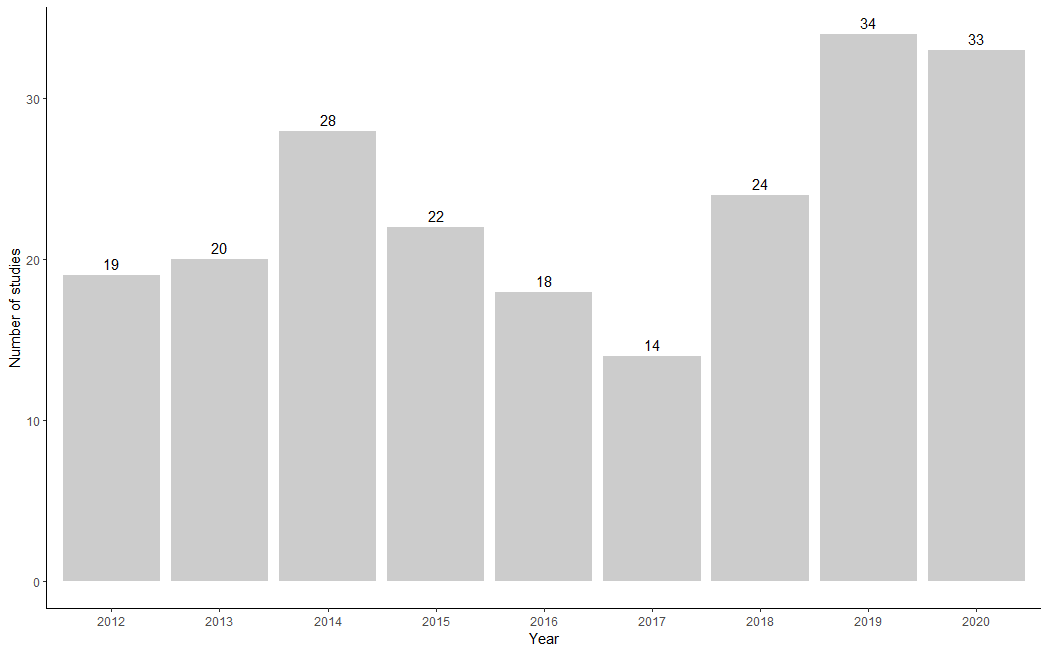


# eFig. 2. Allocated fundings per study


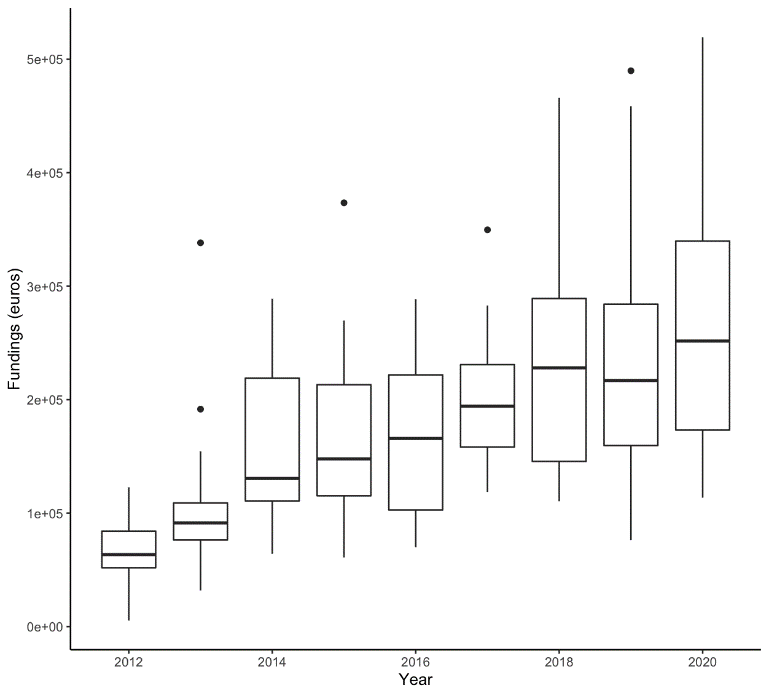


# eFig. 3. Interventional study designs


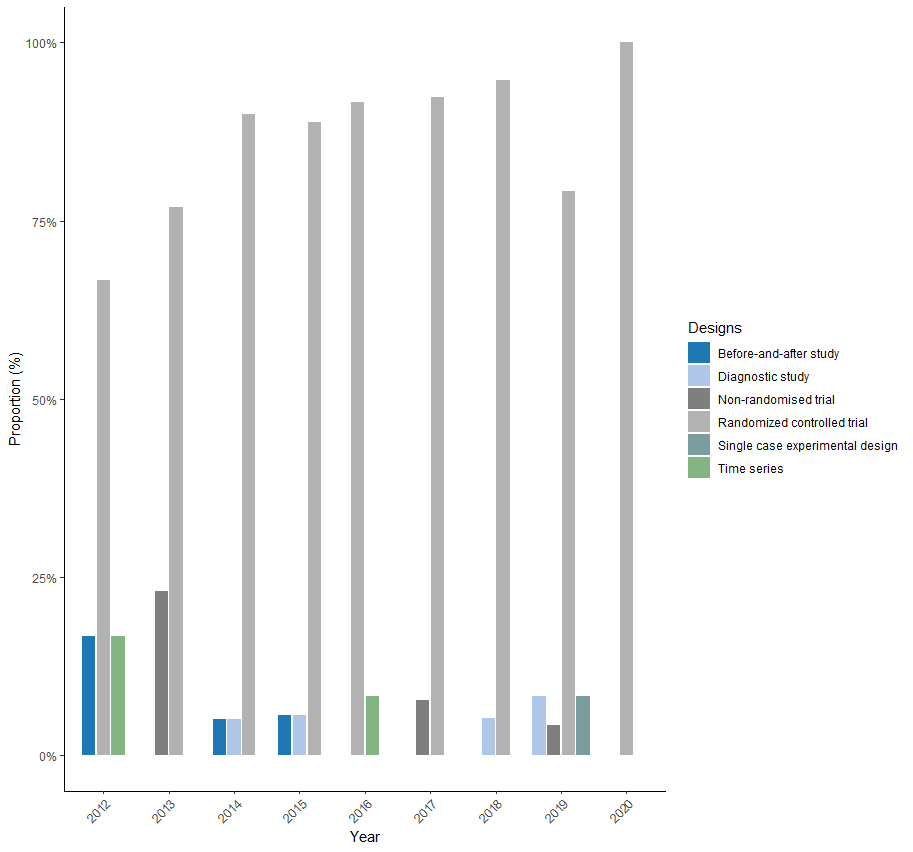


Legend:

The study designs were defined according to the Mixed Methods Appraisal Tool (Hong et al., 2018).

# eFig. 4. Observational study designs


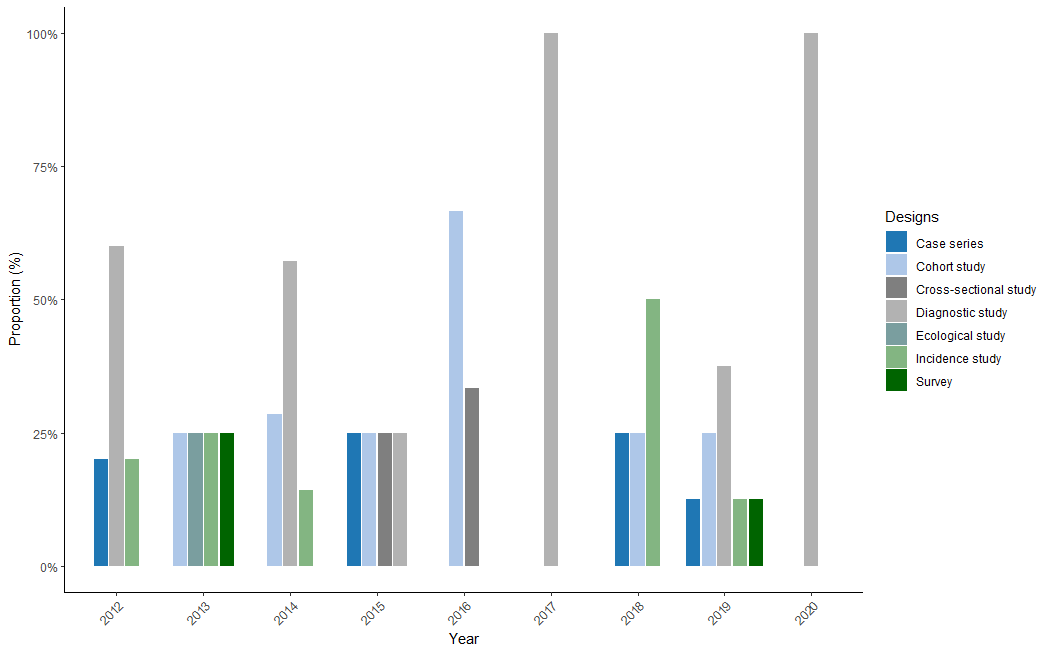


Legend:

Designs were defined according to the Mixed Methods Appraisal Tool (Hong et al., 2018).

# eFig. 5. Proportion of grant recipients among the mean workforce identified over the period in the nursing or allied health professions


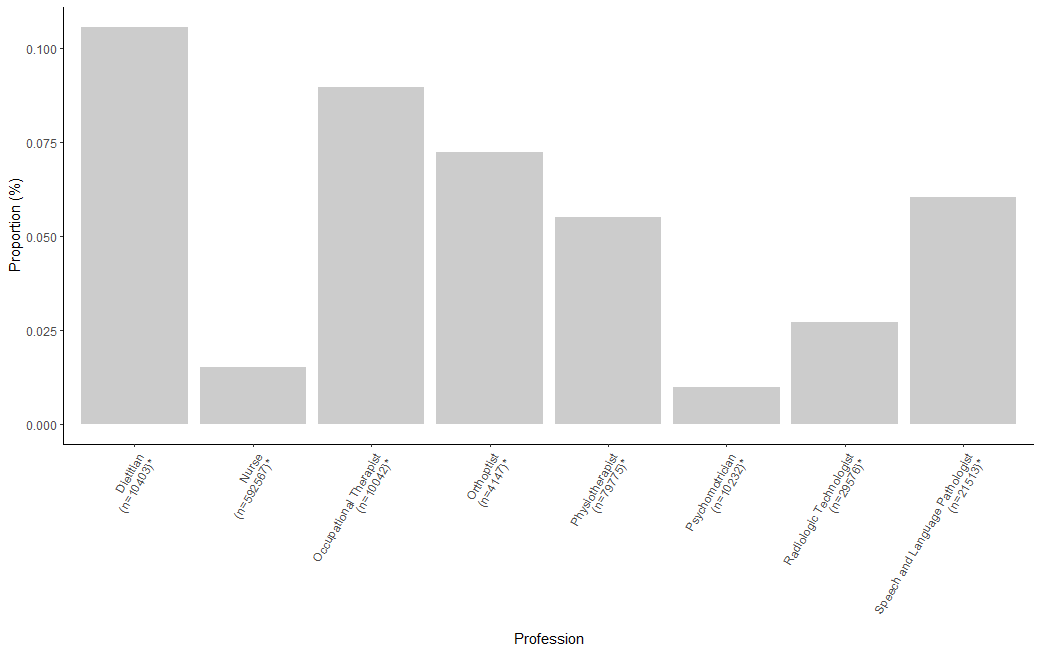


Legend:

Mean workforce on the study period (DREES, 2022)

| **Profession of the grant recipient** | **Academic background of the grant recipien** | **Region** | **Method*** | **Study design*** | **Randomisation** | **Blinding** | **Study status** | **Number of sites** | **Temporality** |
| --- | --- | --- | --- | --- | --- | --- | --- | --- | --- |
| 1. Nurse | MSc | Auvergne-Rhône-Alpes | Quantitative observational | Cohort study | Individual | Quadruple | Ongoing | Monocentric | Prospective |
| 1. Physiotherapist | PhD | Bourgogne-Franche-Comté |  | Case-control study | Cluster | Triple | Completed | Multicentric | Retrospective |
| 1. Podiatrist |  |  |  | Other designs with concurrent group comparison |  |  |  |  |  |
| 1. Occupational therapist |  | Bretagne |  | Cross-sectional study (descriptive or analytical) | Cluster cross-over | Double | Ended |  |  |
| 1. Psychometrician |  | Centre-Val de Loire |  | Incidence study | *Cluster stepped-wedge*** | Simple | Inconnu |  |  |
| 1. Speech and language pathologist |  | Corse |  | Case series |  | Open |  |  |  |
| 1. Orthoptist |  | Grand Est |  | Case report |  |  |  |  |  |
| 1. Radiologic technologists |  | Hauts-de-France |  | Survey |  |  |  |  |  |
| 1. Medical technologist |  | Ile-de-France |  | Ecological study |  |  |  |  |  |
| 1. Hearing aid practitioner |  | Normandie |  | Before-and-after study |  |  |  |  |  |
| 1. Optician |  | Nouvelle-Aquitaine |  | Times series |  |  |  |  |  |
| 1. Prosthetists |  |  |  | *Diagnostic study*** |  |  |  |  |  |
| 1. Dietitian |  | Occitanie | Quantitative interventional | Randomised controlled trial (individual or cluster *or cluster stepped-wedge***) |  |  |  |  |  |
| 1. Physician |  | Pays de la Loire |  | or randomised cross-over study (individual *or cluster cross-over***) |  |  |  |  |  |
| 1. Psychologist |  | Provence-Alpes-Côte d'Azur |  | Non-randomised trial |  |  |  |  |  |
| 1. Other |  | Guadeloupe |  | Non-randomised cross-over design |  |  |  |  |  |
|  |  | Martinique |  | Before-and-after study |  |  |  |  |  |
|  |  | Guyane |  | Times series |  |  |  |  |  |
|  |  | La Réunion |  | *Single Case Experimental Design*** |  |  |  |  |  |
|  |  | Mayotte |  | *Trials Within Cohorts*** |  |  |  |  |  |
|  |  |  |  | *Randomised controlled trial based on health data*** |  |  |  |  |  |
|  |  |  |  | *Diagnostic study*** |  |  |  |  |  |
|  |  |  | Qualitative | Narrative research |  |  |  |  |  |
|  |  |  |  | Phenomenology |  |  |  |  |  |
|  |  |  |  | Grounded theory |  |  |  |  |  |
|  |  |  |  | Ethnography |  |  |  |  |  |
|  |  |  |  | Case study |  |  |  |  |  |
|  |  |  |  | Qualitative description |  |  |  |  |  |
|  |  |  |  | Interpretive description |  |  |  |  |  |
|  |  |  | Mixed | Convergent model |  |  |  |  |  |
|  |  |  |  | Sequential exploratory model |  |  |  |  |  |
|  |  |  |  | Sequential explanatory model |  |  |  |  |  |

# eTable 1. Predefined list of items for data extraction and response modalities

* Methods and study designs were defined according to the Mixed Methods Appraisal Tool (Hong et al., 2018).

** Study designs were added for the present study. Diagnostic study design can be found in both interventional and observational approaches.

| **Characteristics** | **N (%)** |
| --- | --- |
| **Position of the grant recipient in the register data** | **N = 163** |
| Grant recipient named as coordinating investigator | 65 (39.9%) |
| Grant recipient named as scientific director | 8 (4.9%) |
| Names specified are not those of the grant recipient | 40 (24.5%) |
| Not available | 50 (30.7.%) |
| **Studies (overall) and associated publications** | **N = 212** |
| Publication of findings | 18 (8.5%) |
| Publication of the protocol | 6 (2.8%) |

| **Studies completed and associated publications** | **N = 50** |
| --- | --- |
| Publication of findings | 18 (36%) |

| **Publication area** | **N = 18** |
| --- | --- |
| Nursing | 7 (38.9%) |
| Anesthesiology | 2 (11.1%) |
| Medicine, general & internal | 2 (11.1%) |
| Pediatrics | 2 (11.1%) |
| Respiratory system | 2 (11.1%) |
| Critical care medicine | 1 (5.6%) |
| Nutrition & dietetics | 1 (5.6%) |
| Ophthalmology | 1 (5.6%) |
| **Author rank of the grant recipient** | **N = 18** |
| First | 10 (55.6%) |
| Last | 1 (5.6%) |
| Second | 3 (16.7%) |
| Other rank | 2 (11.1%) |
| Absent | 2 (11.1%) |

# eTable2. Additional characteristics of the studies

# References

DREES. (2022) *Démographie des professionnels de santé. DREES Direction de la recherche, des études, de l'évaluation et des statistiques*. Available <https://drees.shinyapps.io/demographie-ps/>. Published: January 2022. Accessed date: 31/10/2023.

Hong, Q. N., Fàbregues, S., Bartlett, G., Boardman, F., Cargo, M., Dagenais, P., Gagnon, M.-P., Griffiths, F., Nicolau, B., O’Cathain, A., Rousseau, M.-C., Vedel, I. & Pluye, P. (2018) *Mixed Methods Appraisal Tool (MMAT), version 2018*. Registration of Copyright (#1148552), Canadian Intellectual Property Office, Industry Canada. Available <http://mixedmethodsappraisaltoolpublic.pbworks.com/w/page/24607821/FrontPage>. Published: March 2013. Accessed date: 17/04/2024.
